# Information gain in the brain's resting state: A new perspective on autism

**DOI:** 10.3389/fninf.2013.00037

**Published:** 2013-12-24

**Authors:** José L. Pérez Velázquez, Roberto F. Galán

**Affiliations:** ^1^Neuroscience and Mental Health Programme, Division of Neurology, Hospital for Sick ChildrenToronto, ON, Canada; ^2^Institute of Medical Science and Department of Paediatrics, Brain and Behaviour Centre, University of TorontoToronto, ON, Canada; ^3^Department of Neurosciences, School of Medicine, Case Western Reserve UniversityCleveland, OH, USA

**Keywords:** brain's resting state, Asperger's syndrome, functional connectivity, stochastic input, relative entropy

## Abstract

Along with the study of brain activity evoked by external stimuli, an increased interest in the research of background, “noisy” brain activity is fast developing in current neuroscience. It is becoming apparent that this “resting-state” activity is a major factor determining other, more particular, responses to stimuli and hence it can be argued that background activity carries important information used by the nervous systems for adaptive behaviors. In this context, we investigated the generation of information in ongoing brain activity recorded with magnetoencephalography (MEG) in children with autism spectrum disorder (ASD) and non-autistic children. Using a stochastic dynamical model of brain dynamics, we were able to resolve not only the deterministic interactions between brain regions, i.e., the brain's functional connectivity, but also the stochastic inputs to the brain in the resting state; an important component of large-scale neural dynamics that no other method can resolve to date. We then computed the Kullback-Leibler (KLD) divergence, also known as information gain or relative entropy, between the stochastic inputs and the brain activity at different locations (outputs) in children with ASD compared to controls. The divergence between the input noise and the brain's ongoing activity extracted from our stochastic model was significantly higher in autistic relative to non-autistic children. This suggests that brains of subjects with autism create more information at rest. We propose that the excessive production of information in the absence of relevant sensory stimuli or attention to external cues underlies the cognitive differences between individuals with and without autism. We conclude that the information gain in the brain's resting state provides quantitative evidence for perhaps the most typical characteristic in autism: withdrawal into one's inner world.

## Introduction

Neuroscience has traditionally focused on the investigation of stimulus-induced activity, whereas spontaneous activity has been considered as noise or background activity of little consequence. However, this view is rapidly changing due in part to empirical evidence indicating the fundamental importance of background, “noisy” activity in the brain for the processing of sensory inputs. Indeed, the brain never rests, for it is constantly receiving inputs, either from the outside or from the body, and even in periods of slow wave sleep the thalamocortical networks display important, coordinated activity. Even when external sensory stimuli are minimized, as in sensory deprivation experiments, the brain responds creating its own world of hallucinations (Sireteanu et al., 2008). Thus, cognitive states in the “idle” brain are not passive and perhaps represent the best opportunity to study the functional connectivity (a term much used these days and perhaps many times abused) of the brain (Galán, 2008; Ringach, 2009; Papo, 2013).

There is a current debate in the autism field about the possible differences in brain connectivity that manifest in the special cognitive style of autistic individuals. In particular, it has been argued that autistic brains are more “disconnected” than those individuals without autism, notion derived mainly form metabolic brain measures like PET or fMRI (Herbert, 2005; Kennedy and Courchesne, 2008; Monk et al., 2009; Thai et al., 2009). Distinct patterns of synchronization of electroencephalographic or magnetoencephalographic (MEG) signals between individuals with and without autism spectrum disorder (ASD) have also been reported (Murias et al., 2007; Pérez Velázquez et al., 2009; Tsiaras et al., 2011; Teitelbaum et al., 2012). As for anatomical features that could underlie possible differences in functional connectivity and thus brain coordination dynamics, alterations in the frontal cortex have been noted in autism, and particularly, an abnormal spatial organization in the microglial-neuronal components (Morgan et al., 2012). Recent studies with difusion tensor imaging have also revealed white matter abnormalities in autism, in particular, a possible atypical lateralization in some white matter tracts of the brain and a possible atypical developmental trajectory of white matter microstructure in persons with ASD (Travers et al., 2012).

Recently, based on the notion that brain activity at rest can be accurately described using stochastic linear dynamics, we used a multivariate Ornstein-Uhlenbeck process (mOUP) to investigate brain dynamics from MEG recordings in ASD and non-ASD individuals (García Domínguez et al., 2013). This method allowed us to estimate not only the functional connections at the sensor level but also the inputs driving the network. Functional connections account for the covariance and lagged correlations between signals recorded from different areas. Inputs reflect contributions to the variance of the recorded signals (outputs) that are not accounted for by the covariance with other signals in the network of sensors. Our results indicated that the dominant connectivity change in ASD relative to controls shows enhanced functional excitation between frontal and parietal/occipital areas. Moreover, the stochastic inputs driving the background activity in the resting state showed a greater spatial homogeneity in ASD than in control individuals, and indeed the spatial complexity of the background noise was significantly lower in ASD subjects. We speculated that higher long-range spatial correlations in the background noise may result from less specificity (or more promiscuity) of thalamo-cortical projections (García Domínguez et al., 2013). All these observations suggest that it may not be a matter of less connectivity in autism, but of changes in connectivity between specific areas as well as in the inputs. As a note of caution, one must bear in mind that in the aforementioned studies with MEG, PET, or fMRI the complex relation between macroscopic recordings and the underlying neuronal activity remains to a certain extent undetermined, so “connectivity” changes are to be understood in a functional rather an anatomical or physiological sense.

The differences found in previous studies on brain coordination dynamics in ASD suggest that information processing/production could be different as well, for it is the coordinated activity of transiently formed neuronal assemblies that underlie information processing and cognition (Flohr, 1995; Bressler and Kelso, 2001; Kelso, 2008; Pérez Velázquez and Frantseva, 2011). Thus, in this study we investigated whether the production of information in periods of little sensory perturbation (resting state) could differ between individuals with and without ASD. As a measure for information production we used the Kullback-Leibler divergence (KLD) between the brain's inputs and outputs. The KLD is also known as information gain or relative entropy (Ihara, 1993) and quantifies differences between two distributions. In our case, the distributions are the probability density of the stochastic inputs driving the brain's activity and the probability density of the brain's activity itself, as recorded with MEG (outputs). We found an increased divergence in children with ASD compared to controls in the resting conditions in which the MEG recordings were taken, and conjecture that this enhanced information gain could be related to one of the most typical characteristics in autism as described already in the early days of autism research: the withdrawal into one's inner world.

## Methods

### Participants and magnetoencephalographic recordings

Data were drawn from a larger sample of children enrolled in previous studies (Pérez Velázquez et al., 2009; Teitelbaum et al., 2012; García Domínguez et al., 2013). In total, MEG data from 19 children, 9 with Asperger's syndrome and 10 age-matched control children, were analyzed. Age range was between 6 and 14 years for the controls (mean: 11.2 years; standard deviation: 2.6 years) and between 7 and 16 for ASD (mean: 10.8; standard deviation: 3.5). The 9 children with Asperger's syndrome were males while the 10 controls were 6 males and 4 females. We note, however, that boys and girls in the control group were not different from each other in terms of our analysis, as shown in our previous study (García Domínguez et al., 2013). The children's parents provided written consent for the protocol approved by the Hospital for Sick Children Research Ethics Board. Participants, who were evaluated by the psychologists in the Autism Research Unit of the Hospital for Sick Children or were recruited from the Geneva Center for Autism and Autism Ontario, met the criteria for ASD based on DSM-IV. Age-matched control children had no known neurological disorders.

MEG recordings were acquired at 625 Hz sampling rate, DC-100 Hz bandpass, third-order spatial gradient noise cancellation using a CTF Omega 151 channel whole head system (CTF Systems Inc., Port Coquitlam, Canada). Out of the 151 sensors, we discarded 10 that were not comparable across all patients due to artifacts or a very low signal-to-noise ratio. Our analysis thus focused on the recordings from the remaining 141 sensors in all patients. Subjects were tested supine inside the magnetically shielded room. Head movement was tracked by measuring the position of three head coils every 30 ms, located at the nasion, left and right ear, and movements less than 5 mm were considered acceptable. Children were instructed to remain at rest during the recording session that lasted between 30 and 60 s. To facilitate the involvement of the children in the experiment and minimize distraction, they were asked to press a button at will with their right hand a few times during the recording session. For each child, an epoch of 30 s was taken off for analysis of functional brain connectivity. All children were awake and had their eyes open during the experiment. Eye-blinking and muscular artifacts have a much larger amplitude than brain activity and are highly correlated across sensors, so they can be easily identified and removed using a well-established approach based on a principal component analysis (Mitra and Pesaran, 1999). In particular, since the artifacts appear in the first few principal components exclusively, they are efficiently cleaned out by removing those components.

### Model of functional connectivity and background noise

In the resting state, the non-linear dynamics of the brain reduces to noise-driven fluctuations around a state of equilibrium, which corresponds to a stable fixed point in neural-mass models of brain dynamics that include conduction delays, dendritic integration and non-linear firing characteristics of neurons (Robinson et al., 1998, 2001). The presence of background noise does not allow the system to quench at the fixed point but perturbs the system in a continuous manner, so that it fluctuates around the equilibrium (Galán, 2008). Thus, consistent with the approach used by several authors (Tononi et al., 1999; Sporns et al., 2000; Galán, 2008; Barnett et al., 2009; Steinke and Galán, 2011; García Domínguez et al., 2013), large-scale spontaneous brain activity is accurately described as a linear stochastic process that is formally equivalent to a mOUP.

(1) $$x_{i}(t+dt)=x_{i}(t)+dt\sum_{j=1}^{N}W_{ij}x_{j}(t)+\eta_{i}(t+dt),$$

where *W*_*ij*_ is the functional connectivity matrix, i.e., the coupling between the *j*-th and the *i*-th nodes; *x*_*i*_(*t*) is the neural activity of the *i*-th node with respect to baseline, measured as the signal from the *i*-th MEG channel at time *t*; η_*i*_ are the residuals (background, uncorrelated white noise) of the *i*-th channel; *N* is the number of nodes (sensors) and *dt* is the sampling interval (1.6 ms). The sign of *W*_*ij*_ represents *functional* excitation (+) or inhibition (−) and should not be confused with excitatory or inhibitory synaptic connections at the cellular level. At the macroscopic level of MEG recordings, functional excitation and inhibition between nodes result from a combined effect of myriad processes, including multiple synaptic interactions and action potentials, which cannot be resolved. The units of *W*_*ij*_ are reciprocal of time, i.e., frequency units.

The functional connectivity matrix *W*_*ij*_ can be obtained from the empirical data *x*_*i*_(*t*) with the Yule-Walker method for multivariate time series (Priestley, 2001). First, equation (1) is written in vector notation as

(2) $$\vec{x}(t+dt)=\vec{x}(t)+W\vec{x}(t)dt+\vec{\eta }(t+dt).$$

Multiplying from the right by $\vec{x}$(*t*)^*T*^ and averaging in time, denoted by brackets 〈…〉, one has *C*_+_ = (*I* + *Wdt*)*C*, with *C*_+_ = 〈$\vec{x}$(*t* + *dt*)$\vec{x}$(*t*)^*T*^〉, *C* = 〈$\vec{x}$(*t*)$\vec{x}$(*t*)^*T*^〉, and *I* being the identity matrix. After computing *C*and *C*_+_ from the recordings, the connectivity matrix is then given by

$$W=\left(C_{+}-C\right)C^{-1}/dt,$$

where *C*^−1^ is the inverse of *C*, or its pseudo-inverse if it is rank-deficient. Once *W* has been determined, the background noise driving the network η_*i*_(*t*) can also be obtained from (2), and their covariance is computed as *Q* = 〈$\vec{\eta }$(*t*)$\vec{\eta }$(*t*)^*T*^〉. Note that the signals *x*_*i*_(*t*) in the resting state have a stable mean (which is negligible relative to the standard deviation), as shown in Figures 2A,B for three arbitrary sensors. For system (2), the covariance matrices of the inputs and outputs are related via (Gardiner, 2004)

(3) $$Q=-dt\left(W^{T}C+CW\right),$$

which allows one to compute *Q* directly from *C* and *W*. This provides a reality check for model (2): the closer the entries in *Q* are to the entries in matrix 〈$\vec{\eta }$(*t*)$\vec{\eta }$(*t*)^*T*^〉, the more accurate is model (2). In our data set, the correlation coefficient between the entries in both matrices is *r* > 0.99 (García Domínguez et al., 2013).

A multivariate Gaussian distribution of variable $\vec{u}$ ∈ ℝ^*N*^ with mean $\vec{m}$ = 〈$\vec{u}$(*t*)〉, and covariance Σ = 〈($\vec{u}$(*t*) − $\vec{m}$)($\vec{u}$(*t*) − $\vec{m}$)^*T*^ 〉 ∈ ℝ^*N* × *N*^ is given by

(4) $$G(\vec{u};\vec{m},\Sigma )\equiv \frac{1}{{\left(2\pi \right)}^{N/2}{\left|\Sigma \right|}^{1/2}}\exp\left(-\frac{1}{2}{\left(\vec{u}-\vec{m}\right)}^{T}\Sigma^{-1}\left(\vec{u}-\vec{m}\right)\right),$$

where |…| denotes the determinant of the matrix inside, or the pseudo-determinant, if the matrix is rank-deficient. For mOUP, the stationary distributions of $\vec{x}$and $\vec{\eta }$ are the multivariate Gaussians, *G*($\vec{x}$; $\vec{0}$, *C*) and *G* ($\vec{\eta }$; $\vec{0}$, *Q*), respectively.

### Entropy and information gain

We computed the entropy of the inputs as the entropy of the distribution of $\vec{\eta }$and the entropy of the output, as the entropy of the distribution of $\vec{x}$. To this end, we recall that the entropy of a multivariate Gaussian distribution (4) with zero mean is given by

(5) $$\begin{array}{l} H\left(\vec{u}\right)=\int_{-\infty }^{\infty }G(\vec{u};\vec{0},\Sigma )\ln G(\vec{u};\vec{0},\Sigma )du^{N}=\frac{1}{2}\ln\left|2\pi e\Sigma \right|\\ \;=\frac{N}{2}\left(1+\ln(2\pi )\right)+\frac{1}{2}\ln\left|\Sigma \right|. \end{array}$$

So that the entropy of the inputs in (2) is *H* ($\vec{\eta }$) = 0.5 · ln |2π*eQ*| and the entropy of the outputs is *H* ($\vec{x}$) = 0.5 · ln |2π*eC*|.

The KLD of two distributions, also known as the relative entropy or information gain, measures how much variability of a stochastic variable $\vec{u}$ ∈ ℝ^*N*^ with distribution *P* cannot be accounted for by a reference distribution *Q*. It is defined as

$$D\left(P||Q\right)=\int_{-\infty }^{\infty }P(\vec{u})\ln\frac{P(\vec{u})}{Q(\vec{u})}du^{N}.$$

To determine the information gain of a mOUP we computed.

(6) $$\begin{array}{l} D\left(G(\vec{x};\vec{0},C)||G(\vec{\eta };\vec{0},Q)\right)=\int_{-\infty }^{\infty }G(\vec{u};C)\ln\frac{G\left(\vec{u};\vec{0},C\right)}{G\left(\vec{u};\vec{0},Q\right)}du^{N}\\ \;=\frac{1}{2}\left(\text{trace}\left(Q^{-1}C\right)-\ln\frac{\left|C\right|}{\left|Q\right|}-N\right). \end{array}$$

The units of the outcome from expressions (5) and (6) are nats. We converted those values to bits by dividing by ln(2), and again by eight to obtain the final result in bytes.

### Invariance of information gain

An important property of the information gain is that it is invariant under linear transformations. This implies that the “cross-talk” or mixing of independent source signals does not affect the information gain. In other words, the information gain measured at the sensor level is the same as the information gain at the source level. The mathematical proof is as follows. Recall that model (2) represents the signal model at the sensor level. Let *U* denote a linear transformation that “unmixes” the sensor level signals $\vec{x}$(*t*) to obtain the source level signals, $\vec{y}$(*t*), so that, $\vec{y}$(*t*) = *U*$\vec{x}$(*t*). In particular, matrix *U* can be computed with an independent component analysis. At the source level, model (2) is transformed into

$$\vec{y}(t+dt)=\vec{y}(t)+V\vec{y}(t)dt+\vec{\xi }(t+dt)$$

with *V* = *UWU*^−1^ and $\vec{\xi }$(*t*) = *U*$\vec{\eta }$(*t*). The covariance matrix of $\vec{y}$(*t*) is then given by *UCU*^−1^ and the covariance matrix of $\vec{\xi }$(*t*) by *UQU*^−1^. The information gain at the source level is thus

$$D_{\text{source}}=\frac{1}{2}\left(\text{trace}\left({\left(UQU^{-1}\right)}^{-1}UCU^{-1}\right)-\ln\frac{\left|UCU^{-1}\right|}{\left|UQU^{-1}\right|}-N\right).$$

We first note that

$$\text{trace}\left({\left(UQU^{-1}\right)}^{-1}UCU^{-1}\right)=\text{trace}\left(UQ^{-1}CU^{-1}\right)=\text{trace}\left(Q^{-1}C\right),$$

due to the invariance of the trace under similarity transformations. We also note that, since the determinant of the product is the product of the determinants one has

$$\frac{\left|UCU^{-1}\right|}{\left|UQU^{-1}\right|}=\frac{\left|U\right|\left|C\right|\left|U^{-1}\right|}{\left|U\right|\left|Q\right|\left|U^{-1}\right|}=\frac{\left|C\right|}{\left|Q\right|}.$$

Thus, the information gain at the source level becomes

$$D_{\text{source}}=\frac{1}{2}\left(\text{trace}\left(Q^{-1}C\right)-\ln\frac{\left|C\right|}{\left|Q\right|}-N\right),$$

which is identical with the information gain at the sensor level (6).

## Results

**Figure 1:**
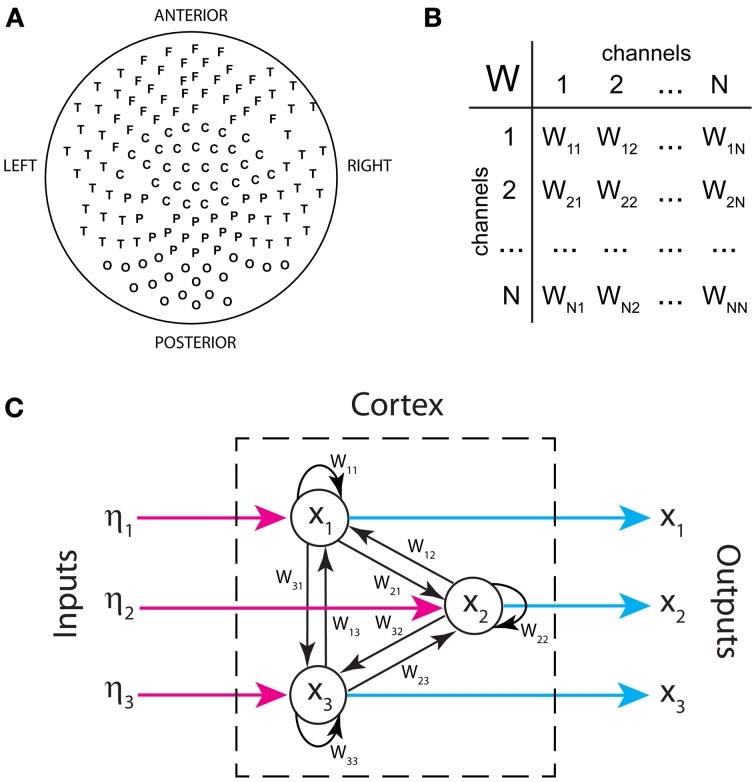
**Inputs and outputs of the cortical network as defined in our analysis. (A)** Spatial arrangement of MEG sensors recording brain activity from the scalp (outputs). **(B)** Functional connectivity as a table of interactions between signals recorded by sensor pairs. **(C)** Schematic representation of the brain's functional connectivity, its inputs and outputs. Only three nodes are shown.

**Figure 2:**
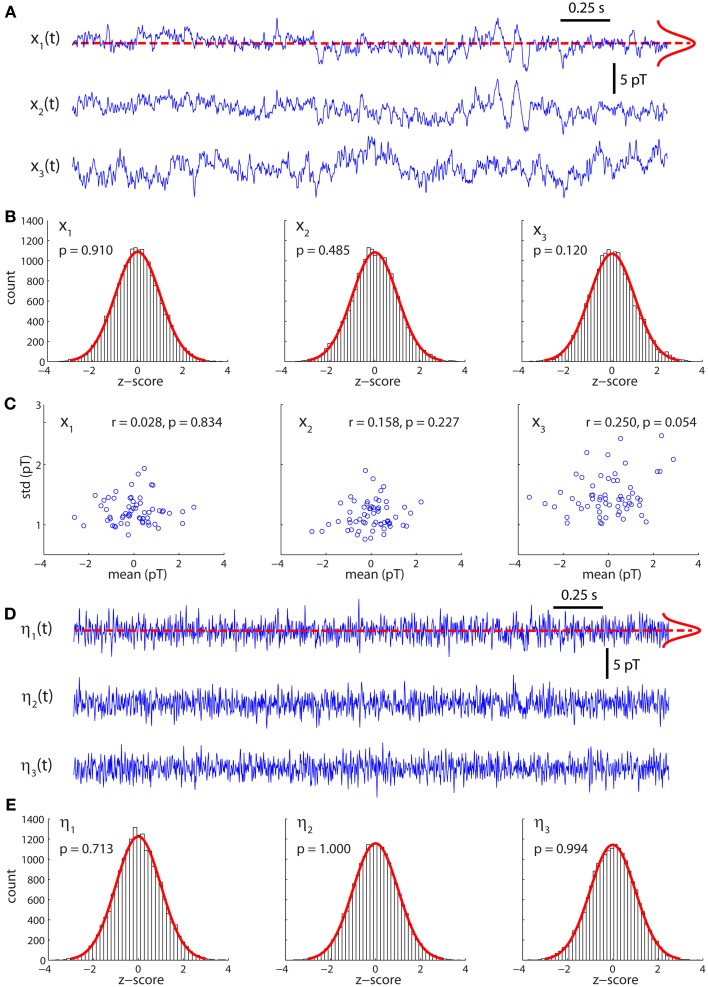
**Activity fluctuations at rest are normally distributed. (A)** Recordings of ongoing activity (3 s long) from three arbitrary sensors in a control subject. **(B)** Activity fluctuations have zero mean and are normally distributed. The histograms were built from segments of 30 s. **(C)** Over short segments of the time series (500 ms long) the mean and standard deviation are uncorrelated, consistently with the assumption of additive noise. **(D)** Residuals (inputs) of the model for the traces shown in **(A)**. **(E)** The residuals are also normally distributed.

Figure 1A displays the arrangement of MEG sensors over the scalp. We only show the positions of the 141 out of 151 sensors that were used in all the subjects (as indicated in methods, 10 sensors were left out due to artifacts and/or low signal-to-noise ratios in different patients). Thus, the dimensions of the functional brain connectivity matrix for each subject are 141 × 141. The sensors cover the occipital (O), frontal (F), central (C), parietal (P), and temporal (T) areas. Each ordered pair of sensors (*i*, *j*)defines an entry in the connectivity matrix *W*_*ij*_ (Figure 1B), which is obtained from the data using model (1). Because MEG signals are most sensitive to cortical activity due to the pronounced decay of magnetic fields with distance, matrix *W*_*ij*_ mainly represents functional connections between cortical areas. A thorough analysis of the connectivity matrices and their differences in ASD was presented in our previous study (García Domínguez et al., 2013). Model (1) also allows one to obtain the inputs to the network, η_*i*_(*t*) as explained in *Methods*. Figure 1C schematically shows the black-box interpretation of the brain dynamics described by equation (1), for just three nodes. The stochastic inputs (background noise), η_*i*_(*t*) impinge on the nodes of the network, which in turn affect each other's activity rate, *dx*_*i*_(*t*)/*dt* according to the connectivity matrix *W*_*ij*_. This determines the instantaneous activity fluctuations (outputs) recorded from each node, *x*_*i*_(*t*). Figure 2A shows traces of ongoing activity recorded with three arbitrary sensors from one of the children. Only 3 seconds of the total recording (30 s) are shown. Traces *x*_1_ and *x*_2_ clearly display correlated fluctuations between them but not with *x*_3_. Figure 2B shows the histograms of the fluctuations recorded from each of those three sensors. The fluctuations around the mean were normalized to the standard deviation of the traces so that the normalized amplitude is given by the z-score. Clearly, the fluctuations are normally distributed, as demonstrated by the excellent fit to a Gaussian (red line). The high *p*-values confirm the null hypothesis of the chi-square goodness-of-fit test, namely, that the fluctuations have a normal distribution in each sensor.

Model (1) assumes that the noise is additive and hence state independent. In such a case, the mean and variance of small segments of the time series should be independent of each other. To test this, we divided the traces in successive segments of 500 ms and plotted the mean over each segment against its standard deviation (Figure 2C). For all traces, the Pearson's correlation coefficient was not statistically significant, suggesting that both quantities are indeed independent of each other.

Figure 2D shows the stochastic inputs to the three nodes investigated above, η_1_, η_2_ and η_3_. Compared to the outputs in Figure 2A, the inputs display no significant temporal structure and lower amplitudes, which is what one would expect for the residuals of a parametric model, such as model (1). Figure 2E shows that the inputs are also normally distributed.

**Figure 3:**
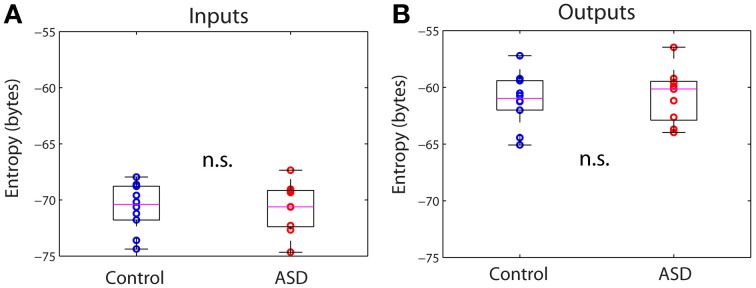
**Differential entropies of the inputs and outputs. (A)** Inputs. **(B)** Outputs. There are no significant differences between groups in either case.

From the connectivity matrix, *W*_*ij*_ and the covariance matrix of the signals, *C*_*ij*_, one can readily obtain the covariance matrix of all the inputs, *Q*_*ij*_, using formula (3) in *Methods*, without having to determine them explicitly. This allows us to efficiently compute information theoretical measures. Figure 3 shows the entropy of the inputs and outputs for the control and ASD groups. The entropy is larger for the outputs than for the inputs for both groups. However, the differences between both groups for inputs or outputs are not significantly different (*p* >> 0.05, Wilcoxon rank-sum test). We note that the entropy values are negative. Indeed, while entropy values for discrete signals are non-negative, the entropy of continuous signals (differential entropy) may be negative. Negative entropy values result from expression (5), when |2π*e*Σ| < 1. Note that the value of this determinant depends on the units of the covariance, so our choice of those units affects the value of the entropy. Moreover, the entropy for continuous signals is very sensitive to their variance and because the amplitudes of the activity fluctuations are not significantly different between control and ASD (data not shown), neither are the entropies. These are well-known caveats that preclude the interpretation of entropy (or more accurately, differential entropy) as a measure of information content for continuous signals (Ihara, 1993). This contrasts with the case of discrete signals, for which entropy is legitimately interpreted as the expected value of information contained in a signal (Ihara, 1993).

**Figure 4:**
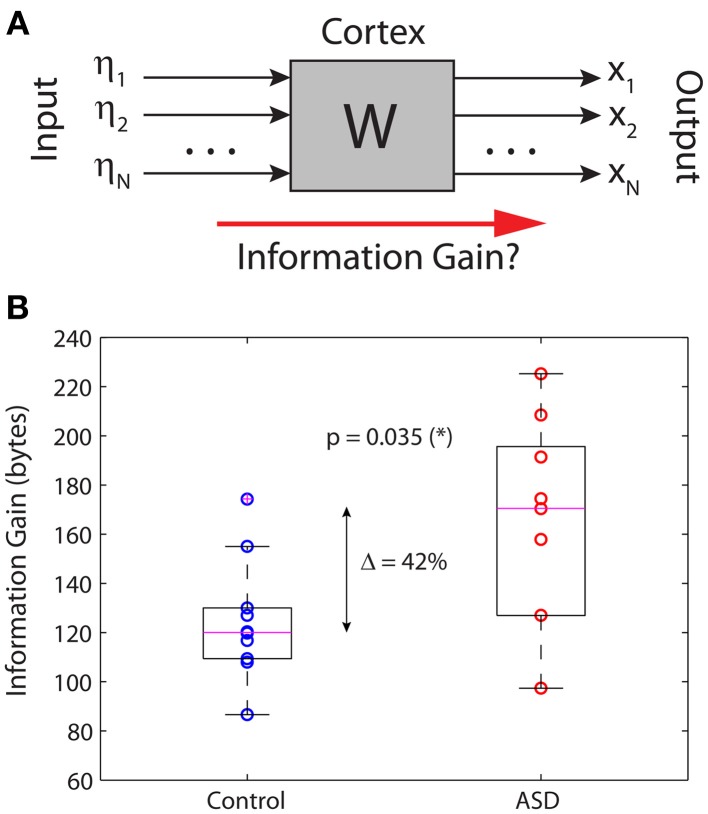
**Information gain in the brain's resting state. (A)** Schematic black-box representation of cortical dynamics in the resting state. **(B)** The information gain is significantly increased by 42% in autistic relative to non-autistic children.

A more relevant measure of information that has the same interpretation and properties for continuous and discrete signals is the relative entropy, or information gain, defined as the KLD between two distributions (see *Methods*). In lay terms, the KLD measures how much variability of a stochastic variable with distribution *P* cannot be accounted for by another stochastic variable with distribution *P*′. This interpretation justifies the alternative name of “information gain” about one variable by knowing the other. In our context, we computed the KLD to *quantify the amount of information of the outputs that cannot be accounted for by the inputs*. In other words, we quantified how much information is “created” by the brain in the resting state. Figure 4A shows a simplified black-box interpretation of the brain, in a similar fashion to Figure 1C but for an arbitrary number of nodes and without paying attention to the details of the brain's network contained in the box. The key finding of this article is shown in Figure 4B, which plots the information gain of the brains in the control and ASD groups. Despite some overlap between the distributions, the medians of both groups are significantly different (Wilcoxon sum-rank test; *p* = 0.035). In particular, the information gain in the ASD group is 42% larger on average, indicating that ASD brains produce more information from the stochastic inputs driving them.

**Figure 5:**
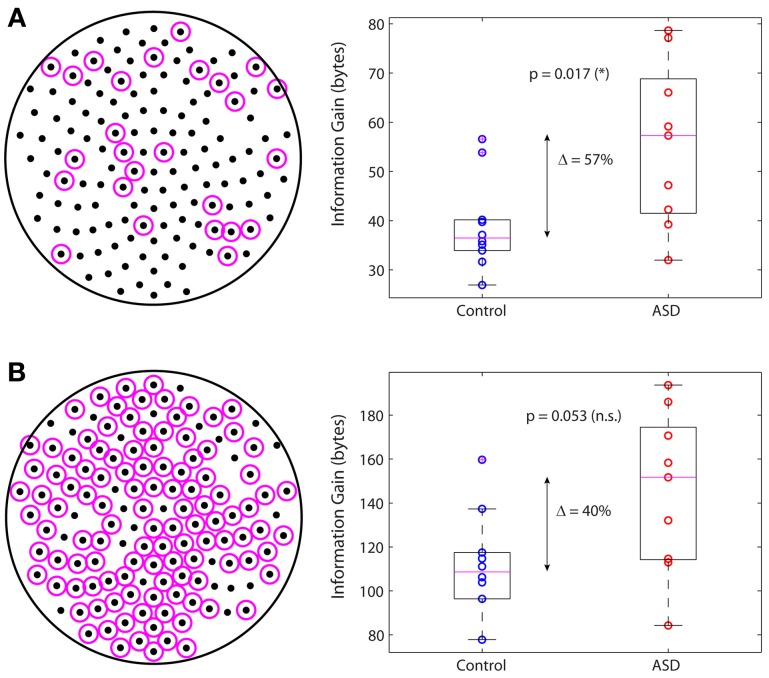
**Main subnetwork contributing to the increased information gain. (A)** Subnetwork containing the largest (in absolute value) and most significant changes in functional connectivity (left). This subnetwork alone accounts for a significant increase in information gain in autism (right). **(B)** Complementary subnetwork containing the remaining sensors (left). This subnetwork cannot account for the change in information gain on its own, as the increase is not statistically significant (right).

In a previous study we identified the subnetwork of sensors containing the functional connections whose changes in autism are largest in absolute value and most significant relative to control (García Domínguez et al., 2013). We then asked whether this subnetwork on its own can account for the increased information gain in autism. Figure 5A displays the sensors belonging to this subnetwork (left; magenta circles) and the information gain for this subnetwork in the control and ASD groups (right). The difference of the medians is 57% and it is statistically significant (*p* = 0.017; Wilcoxon rank-sum test). In contrast, if one considers the complementary network, i.e., the other nodes in the sensors network (Figure 5B, left), the difference of the medians is 40% but not statistically significant, as it falls below the 95% confidence level (*p* = 0.053; Wilcoxon rank-sum test). In conclusion, although all nodes contribute to the information gain, those nodes encompassing the interactions with the largest changes in autism contribute more to the increase in information gain. However, changes in connectivity alone are not sufficient to account for the difference in information gain that we observe in ASD, as the information gain depends not only on *W* via *C*, but also on matrix *Q*, which we know from our previous study that is also significantly different in ASD (García Domínguez et al., 2013). The question then is: do changes in *W* compensate for changes in *Q* or do these changes act synergistically to increase the information gain? Our analysis suggests the latter may be the correct answer, or at least, that changes in connectivity cannot fully compensate for changes in the inputs.

## Discussion

The term autism (from the Greek autos, meaning “self”) was coined in 1911 by Swiss psychiatrist Eugen Bleuler, who used it to describe withdrawal into one's inner world (even though at this time he was referring to schizophrenia patients). Later, other studies defined more precisely the syndrome (Kanner, 1968). The neurophysiological reasons responsible for a certain detachment from the environment of individuals with ASD remain unknown, and several scholars have proposed ideas mostly centered on the psychological level of description. Whereas much brain structural and genetic studies are being done in autism research, the investigation of the brain dynamics is lagging considerably behind. Here, we have explored what the background brain activity in resting conditions (when individuals are not presented with specific sensory stimuli) may reveal about the inner processing of the brain in terms of information production, quantified as relative entropy. Our analysis of MEG signals recorded at rest indicated that the brains of individuals with ASD, Asperger syndrome in this case, produce more information than the age-matched participants with a 42% increase on average. These significant differences cannot be attributed to the gender-ratio mismatch in our cohort. Although there were 6 males and 4 females in the control group, and no females in the ASD group, the control group was fairly homogeneous: there were no significant differences in the information gain between the boys and girls within the control group (*p* = 0.76; Wilcoxon rank-sum test).

We decided to focus on spontaneous brain activity in resting conditions because the fundamental importance of the ongoing, “noisy” nervous system activity is widely recognized today, and a more in-depth investigation of brain activity in periods of minimal sensory perturbation has been advised by several scholars as it may provide the best opportunity to study the intrinsic connectivity of the brain, in the absence of major sensory perturbations (Galán, 2008; Ringach, 2009; Steinke and Galán, 2011; García Domínguez et al., 2013; Papo, 2013). From an analytical perspective, there are two important reasons for investigating brain activity in the resting state. The first one is that in this case, the brain dynamics are described by stochastic model (1), which implies that functional connections (*W*_*ij*_) are constant, in contrast to the stimulated brain, in which interactions between different areas are state-dependent and typically non-linear. The second reason is that in the brain's resting state the stochastic inputs, η_*i*_, as well as the activity fluctuations, *x*_*i*_ (outputs) are normally distributed. Thus, the distributions of $\vec{\eta }$ and $\vec{x}$ are both *N*-dimensional Gaussians. This enables an accurate parametric estimation of the entropies and relative entropy, as shown above. If the fluctuations are not normally distributed, as it is frequently the case for stimulus-evoked activity, a parametric estimation of information theoretical measures is in general not possible. To compute entropies and related quantities in such cases, one needs to estimate the probability densities of the data. However, the estimation of high-dimensional probability densities requires very large datasets, which are virtually impossible to collect in current experimental settings.

In our study, functional connections between areas and their inputs are defined operationally from model (1): functional connections account for the covariance and lagged cross-correlations between signals recorded from different areas, whereas the inputs are defined as contributions to the variance that are not accounted for by the covariance with other signals in the network of sensors. Neither the functional connections nor the inputs represent specific neuronal elements, although they obviously emanate from them in a complex, undetermined manner. Certainly, a multiscale modeling approach, from single cells to neural mass models, is worth attempting. This is, however, a daunting task, as recognized by other authors working on this problem (Deco et al., 2008).

There are two important considerations about the dynamical model used in our study: (1) the suitability of a linear model for large-scale brain dynamics* in the resting state*; and (2) the interpretation of the inputs in the model. As for the first consideration, we note that there is no contradiction between our stochastic linear model and the fact that brain dynamics are strongly non-linear because we do not intend to model neuronal dynamics *per se*. We are rather modeling the recorded signals, which are magnetic fields that do superimpose linearly. An analog dichotomy takes place in weather forecasting: although the dynamics of air masses are turbulent, chaotic and therefore, unpredictable, when considered over a large area the flow of air masses becomes predictable within a time window of a few days. These coarse dynamics of air masses fit very well a linear multivariate stochastic process, which can then be used to accurately forecast variations and co-variations of air pressure and temperature at different locations (Storch and Zwiers, 2001). Similarly, in neural mass models the strong non-linear dynamics of single neurons, when averaged over a fairly large spatial range, display fluctuations around a mean that make a stochastic linear model suitable for the description of large-scale activity. As noted by Nunez and Srinivasan, “the question of brain linearity depends on context and the level […] addressed […]. It is only in mathematics that a sharp distinction exists between linear and non-linear system” (Nunez and Srinivasan, 2006). We also note that non-linear neuronal networks, like those based on the celebrated Wilson-Cowan model and the neural-mass models frequently possess hyperbolic fixed points which are linearly stable. The brain's resting state we record from corresponds to this kind of stable state, as shown in Figures 2A,B, in which baseline activity is characterized by fluctuations around a fixed mean. Indeed, linearization of neural-mass dynamics around a hyperbolic fixed point leads to model (2) when stochastic perturbations are included. Several recent papers have taken advantage of this fact to investigate the link between connectivity and spontaneous activity patterns in a neural network model (Galán, 2008; Barnett et al., 2009; Steinke and Galán, 2011; García Domínguez et al., 2013). Outside the resting state, during sensory stimulation, brain activity typically has a moving baseline, or low frequency modulation of the fluctuations, which results from the non-linear regime of the neural-mass dynamics, and therefore, it is inconsistent with model (2). That is the reason why our model should only be applied to brain activity in the resting state.

As for the interpretation of the inputs in our model, we remark that subcortical structures relay inputs to the cortex and probably more (if these could be quantified) than those from the external sensorium, which with the exception of the olfactory system are filtered through the thalamus. Our model considers both sources of fluctuating inputs together: those coming from the external world and those from internal organs are similar for our purposes because the other organs are, after all, external to the brain too, so they are all just inputs. Regarding this matter of differentiating internal vs. external inputs, we find the thoughts by Nachev and Husain quite appealing; in their words “the contrast between internally and externally-generated actions is empirically intractable” (Nachev and Husain, 2010).

Large-scale recordings, such as MEG traces, have some limitations to keep in mind (Gross et al., 2013). The signals detected by MEG reflect population-scale levels of activity in large neuronal networks. Insights gained from the analysis of MEG data are limited to coarse relationships between large populations of cells rather than the detailed understanding of interactions between individual cells. Moreover, spontaneous activity at any given sensor may contain activity from multiple distributed sources, and conversely, the activity of a single signal source can introduce coordinated changes at multiple sensors. For these reasons, functional connectivity estimated from signals recorded by the sensors does not necessarily reflect the actual connectivity between the brain areas next to where the sensors are located. Thus, a distinction between sensor-level and source-level connectivity is pertinent to MEG but also to all technologies for measuring large-scale brain connectivity that are currently available. Ideally, connectivity analysis should be performed at the source level. However, source reconstruction clearly adds another level of complexity to the analysis and may even yield spurious results, as it is an ill-posed mathematical problem (Gross et al., 2013). This implies that assumptions must be made about the origin and location of the sources in order to properly constrain the solution to the problem. Whereas certain assumptions may be reasonable for stimulus-driven experiments because specific sensory or motor areas are expected to be strongly activated, this is not trivial for ongoing activity where no specific areas are expected to dominate the brain dynamics. Importantly, we have shown here (see *Methods*) that the information gain in the brain's resting state is the same for the source and sensor levels. Thus, the results we report here are unaffected by any possible cross-talk between sensors or mixing of independent source signals at the sensor level.

When addressing queries on information processing in nervous systems, the question of what is meant by “information” always arises. There are several different notions about what information is and represents, and depending on the research field, e.g., thermodynamics, cybernetics, information theory etc., one may come across different definitions. In general terms, however, the concept of information refers to the ability of a given signal to encode a message with a presumed alphabet regardless of its content. That is, the information is agnostic to semantics or meaning. In plain mathematical terms, the information gain used in this study is nothing but a measure of the global differences between the distributions of the input to and output of the brain in its resting state. It therefore quantifies the *degree of transformation of the inputs into the outputs*. Because this transformation is made by the brain's network, the information gain can literally be regarded as the amount of information created by the brain which is not already present in the inputs.

On a more philosophical level, the general expression “brain information processing” is commonly used without specific details as to what this information is, but it serves the purpose as it relies on certain intuitive knowledge that neuroscientists share and accept. If, as Heinz von Foerster declared, “information is a relative concept that assumes meaning only when related to the cognitive structure of the observer (the recipient)” (Von Foerster, 2003), and the activity of the brain cellular circuits is roughly considered as the production of “novel” associations between stimuli (external or internal), then perhaps an increase in the difference between the stochastic input and output, as found in our work, could conceivably be associated with a more pronounced “mental inner life” that, roughly speaking, may result in the common detachment of individuals with ASD from their environment. Perhaps a bit more specifically, following Davies' recent postulate of two types of information in biological systems (Davies et al., 2013), structural and functional, it could be reasoned that in the nervous system the structural information derived from direct anatomical connections between cells is responsible for the maintenance of memory and other specific aspects that need to be maintained in an stable manner, whereas functional information, which is what we measured in our studies, could be related to the rate of cell assembly formation, to the transient establishment of coordinated activity amongst brain cell networks which is the basis of cognition (Bressler and Kelso, 2001; Kelso, 2008; Pérez Velázquez and Frantseva, 2011). As a predecessor of the current conceptualization, Hans Flohr already proposed almost two decades ago that the rate of cell assembly formation determines cognition (Flohr, 1995). A precise investigation of how cell assemblies form and disappear and the relation between these ephemeral brain functional networks and cognitive/psychological aspects is difficult to achieve with current methods in brain and cognitive science. Nevertheless, these types of research encompassing biophysics and psychological observations, we venture, will be a fundamental part of the immediate future of neuroscience research. In fact, with the current theoretical conceptualization of nervous system dynamics based on dynamical bifurcations that switch brain/cognitive states in a flexible manner, it is not surprising that more investigations on the role of background activity in brain information processing are being conducted at several levels of description (Liljenstrom, 1996; Mcmillen and Kopell, 2003; Pérez Velázquez et al., 2007; Zhou et al., 2010; Luczak et al., 2013).

Combining several empirical observations, the picture that emerges is that a tendency toward enhanced excitatory activity in the cell circuitry in the autistic brain (Rubenstein and Merzenich, 2003; Han et al., 2012) results in hyperactivity in certain brain regions (García Domínguez et al., 2013) that in turn enhances the tendency toward increased synchronous activity in those areas, e.g., parietal cortices (Pérez Velázquez et al., 2009; Teitelbaum et al., 2012), which is reflected in greater spatial correlation in the background activity (García Domínguez et al., 2013) and in a more pronounced information production from the background activity, as found in this study. More generally, these related tendencies toward more than normal excitation and synchronization could underlie most of neurological and psychiatric disorders (Pérez Velázquez and Frantseva, 2011; Yizhar et al., 2011). All these neurophysiological differences between autistic and non-autistic brains, we propose, could contribute on the behavioral level to the known withdrawal to their inner world of individuals with autism. While, at this stage, this is a conjecture, it is perhaps useful to start the never easy attempt of framing neurophysiological data into psychological aspects. Our study is intended as an initial step in the investigation of how information generation in the brain relates to cognitive/psychological aspects and our results allow us the following speculations. It is noteworthy that the subnetwork of sensors that significantly contributes to the increased information gain in autism (as shown in Figure 5A) contains a combination of frontal, temporal and parietal areas which also correspond to the default mode network; the brain areas that reduced their activations during processing of external stimuli and are preferentially active when individuals do not focus on the external world (Buckner et al., 2008). Moreover, this subnetwork contains a number of midline sensors: medial frontal, central and parietal (Figure 5A). Remarkably, both the default network and midline brain structures have been proposed to be fundamental regions for self-processing (Northoff and Bermpohl, 2004), and there are numerous studies that reported the association of activation in parietal and medial frontal cortex in self-referential processing (Lou et al., 2004; D'argembeau et al., 2007). Nevertheless, it should be considered that each brain area is “activated” by other connected nets, which means that these regions proposed in the literature, while significantly associated with self-referential processing, receive inputs and integrate their activity with others possibly subcortical areas (Northoff et al., 2011). It is also of interest that distinct patterns of synchronization in the “default areas” have been noted (Fingelkurts, 2011), especially an increase in phase synchrony when subjects attention is internally focused (Kirschner et al., 2012). These previously reported neurophysiological phenomena in those brain areas may contribute to the observed differences between the two groups in the information gain reported in this work, and particularly the higher information gain in the ASD group could therefore be related to the more intense “inner world” that autistic individuals normally have.

Future studies may consider applying our method to other cognitive phenotypes as well. To interpret information gain in other contexts one must bear in mind that it explicitly depends on the inputs and outputs of the resting state network, and implicitly (via the output covariance) on the functional connectivity. Significant changes in information gain must therefore result from changes in at least one of these measures or, as it is the case in our study, in all the three measures. One may then ask whether changes in connectivity tend to compensate for changes in inputs so that the information gain is barely altered, or whether those changes act synergistically to exacerbate alterations in neuronal activity and information gain. Finally, to more explicitly address the relation between information gain and particular psychological traits, it is worth noting that people with schizophrenia are characterized by excessive self-awareness (Frith, 1979), which taken to the limit may lead to hallucinations. We surmise that if our analysis of the brain's resting state were conducted on people with schizophrenia, it would also show a significant increase in information gain that reflects the ability of the brain to generate complex activity on its own, even in the absence of significant stimulation.

### Conflict of interest statement

The authors declare that the research was conducted in the absence of any commercial or financial relationships that could be construed as a potential conflict of interest.
